# Predictive equations over-estimate the resting energy expenditure in amyotrophic lateral sclerosis patients who are dependent on invasive ventilation support

**DOI:** 10.1186/1743-7075-7-70

**Published:** 2010-08-26

**Authors:** Waltteri Siirala, Klaus T Olkkola, Tommi Noponen, Arno Vuori, Riku Aantaa

**Affiliations:** 1 Department of Anaesthesiology, Intensive Care, Emergency Care and Pain Medicine, Turku University Hospital and University of Turku, Kiinamyllynkatu 4-8 FI-20520 Turku, Finland; 2 Turku PET Centre, Turku University Hospital, Kiinamyllynkatu 4-8 FI-20520 Turku, Finland

## Abstract

**Background:**

Amyotrophic lateral sclerosis (ALS) is a form of degenerative motor neuron disease. At the end stage of the disease artificial feeding is often required. Nevertheless, very little is known about the energy demand of those ALS patients who are chronically dependent on tracheostomy intermittent positive pressure ventilation. The objective of our study was to clarify the resting energy expenditure (REE) in mechanically ventilated ALS patients.

**Methods:**

We measured the REE of five ALS patients (four men, one female) twice during a 12 month-period using indirect calorimetry with two sampling flow settings (40 L/min and 80 L/min). The measured REEs (mREE) were compared with values calculated using five different predictive equations.

**Results:**

The mean (± SD) of all mREEs was 1130 ± 170 kcal/d. The measurements with different flow settings and at different time instances provided similar results. The mean of mREEs was 33.6% lower, as compared to the mean calculated with five different predictive equations REE (p < 0.001). Each of the predictive equations over-estimated the REE.

**Conclusions:**

The mREE values were significantly lower for every patient than all the predicted ones. Determination of daily nutrition with predictive equations may therefore lead in mis-estimation of energy requirements. Because ALS patients may live years with artificial ventilation their nutritional support should be based on individual measurements. However, further study is needed due to the small number of subjects.

## Backround

Amyotrophic lateral sclerosis (ALS) is a form of degenerative motor neuron disease of unknown etiology [1,2]. The prevalence is 4-8 cases per 100 000 and the annual incidence is approximately 1-2 per 100 000 [3]. The disease is characterized by progressive muscle weakness and atrophy throughout the body as both the upper and lower motor neurons are degenerated. Although the sequence and rate of emerging symptoms vary from person to person, the patients may eventually lose the ability of all voluntary movements, and become immobilized. As also the diaphragm and intercostal muscles weaken, the patients' ability to breathe spontaneously decreases [1,4]. The median survival without mechanical ventilatory support is approximately 30 months from the date of diagnosis. Death is usually a consequence of severe hypoventilation and pneumonia [5].

Non-invasive ventilation (NIV) is often constituted when respiratory weakness starts to occur [6]. NIV may become insufficient with further progression of the illness and more invasive measures, most often tracheostomy and intermittent positive pressure ventilation (TIPPV), must be taken if the patient and the caregivers agree upon such [7]. Indeed, TIPPV can improve survival artificially even for years in ALS [8,9]. Nevertheless, there are ethical as well as cultural issues associated with the use of invasive respiratory therapy and therefore the incidence of TIPPV in ALS patients has been reported to be 10% or even lower [8,10,11].

The prolonged survival among ALS patients with TIPPV makes their nutritional management challenging. Due to immobility, there may be a risk of positive energy balance if the energy demand is estimated inappropriately. The storage of excessive carbohydrate in fat tissue (lipogenesis) will increase the total body carbon dioxide production which in turn will increase the need for ventilation and thus increase the work of breathing [12,13]. Increased carbon dioxide production may cause respiratory acidosis in these totally ventilator-dependent patients because they cannot increase the ventilation rate and tidal volume themselves. Secondly, overestimation in energy supply can easily lead to obesity and increased risk of diabetes, hypertension, ischemic heart disease, stroke and renal disease [14], and make nursing more difficult [15]. Percutaneous gastrostomy has been recommended as a route of feeding when the oropharyngeal muscles are affected [16]. Yet, little is known about the energy consumption of such patients.

Resting energy expenditure (REE) is the amount of energy needed to maintain the normal body functions at rest, excluding the thermal effect of food and physical activity, whereas total energy expenditure (TEE) is the total amount of energy needed per day including that needed for muscle activity [13]. REE has been calculated to be approximately 0-30% below the TEE in healthy subjects [17]. Indirect calorimetry is a noninvasive method to measure the REE. Measured REE (mREE) is determined from oxygen consumption (VO_2_) and carbon dioxide production (VCO_2_), estimated from breathing gases [18]. The accuracy of this method has been proven as reliable in several studies since these devices were introduced in the 1980's [19-23].

If indirect calorimetry is not available, predictive equations such as the Harris-Benedict [24], the Food and Agriculture/World Health Organisation/United Nation Union (FAO/WHO/UNU) [25], the Owen [26,27], the Mifflin-St Jeor [28] or the Fleisch equation [29] have been used to calculate a predictive REE (pREE). These equations utilize the subject's weight, height, age and/or body surface area. However, little is known about the accuracy of these predictive equations to assess the REE of immobile ALS patients on TIPPV.

Our clinical impression has been that the predictive equations overestimate the energy requirements of patients requiring TIPPV, leading easily to obesity. The objective of this study was to compare predicted REE values with measured indirect calorimetry values in 5 patients with ALS totally dependent upon TIPPV.

## Methods

### Subjects

Five patients (age > 18 years, four males, and one female) with ALS, according to El Escorial World Federation of Neurology criteria [30], were studied as part of their routine clinical care. The study protocol was approved by the local ethics committee. All patients had been permanently dependent on TIPPV for years (mean duration of TIPPV 69 months, range 44-96 months). All patients were treated at home by trained caregivers. Two patients were in a totally locked-in state (i.e. they were not able to perform any voluntary movements) and three patients were in a minimal communication state (i.e. they were able to communicate only with eye movements). For full-time therapy, TIPPV (PLV 100, Respironics, Muryville, USA) was carried out with an open system ventilation technique in which the inspiratory gases were conducted via an inlet tube to the tracheostomy cannula and the expiratory gases were released to room air from a valve near the tracheostomy cannula. Supplemental oxygen was not used.

### Study design

VO_2_, VCO_2_, respiratory quotient (RQ = VO_2_/VCO_2_), and mREE were determined with indirect calorimetry (Deltatrac II metabolic monitor, Datex-Ohmeda, Helsinki, Finland) by the same observer (WS). The measurements were carried out in the patients' home in the morning between 08 am and 11 am at least twice within a 1 - 12 month interval. All the patients were continuously treated with TIPPV throughout the day. Indirect calorimetry was accomplished while the patients were awake and ventilated with TIPPV using ambient air (FiO_2 _21%). No additional oxygen was used during any measurements. The patients were not on any medications one week before or during the measurements. All REE measurements were carried out after 12 hours of fasting. The mREE values were compared to the pREE values calculated using five different equations (Table 1).

**Table 1:** Equations used for the estimation of resting energy expenditure in kcal/d.

Name of the equation	Equation
Harris-Benedict [24]	
Men	66.47 + 13.75 × weight + 5.0 × height - 6.75 × age
Women	655.09 + 9.56 × weight + 1.84 × height - 4.67 × age

WHO/FAO/UNU [25]	
Men	
18 - 30 yrs	15.4 × weight - 27 × height + 717
31 - 60 yrs	11.3 × weight + 16 × height + 901
> 60 yrs	8.8 × weight + 1128 × height - 1071
Women	
18 - 30 yrs	13.3 × weight + 334 × height + 35
31 - 60 yrs	8.7 × weight - 25 × height + 865
> 60 yrs	9.2 × weight + 637 × height - 302

Mifflin-St Jeor [28]	
Men	9.99 × weight + 6.25 × height -4.92 × age + 5
Women	9.99 × weight + 6.25 × height -4.92 × age - 161

Owen [26,27]	
Men	879 + 10.2 × weight
Women	795 + 7.18 × weight

Fleisch [29]	
Men	24 × BSA¹ × (38 - 0.073 × (age - 20))
Women	24 × BSA¹ × (35.5 - 0.064 × (age - 20))

¹BSA = body surface area in m² is calculated according to DuBois & DuBois equation [41].

The weight should be given in kilograms and the height in centimetres in all equations.

### Indirect calorimetry measurement

In Deltatrac II a constant air flow (Q) is drawn into the monitor unit. The device has four different sampling flow values (3, 12, 40 and 80 L/min) for patients at different weight ranges (< 3, 3-20, 20-120 and > 120 kg, respectively) which correlate with the patient's tidal volume. Deltatrac calculates VO_2 _from the measured insipratory and expiratory oxygen concentrations, using the Haldane transformation as follows:

$${\text{VO}}_{2}=(\text{Q}/(1-{\text{FiO}}_{2}))(({\text{FO}}_{2}-({\text{FiO}}_{2})({\text{FCO}}_{2}))$$

and the VCO_2 _from the measured carbon dioxide concentration from expiratory gas as follows:

$${\text{VCO}}_{\text{2}}=\text{ Q}\left({\text{FeCO}}_{\text{2}}-{\text{FiCO}}_{\text{2}}\right)$$

FiO_2 _, FeO_2_, FiCO_2 _and FeCO_2 _are the measured gas concentrations from inspired (i) and expired (e) ventilation gases. FO_2 _is the consumed oxygen calculated as FiO_2 _- FeO_2 _and the FCO_2 _is the produced carbondioxide FeCO_2 _- FiCO_2_. As normal room air contains less than 0.03% of carbon dioxide, FCO_2 _can be considered to be equal to FeCO_2 _[18].

The mREE was calculated from measured VCO_2 _and VO_2 _values using the Weir's equation [31]:

$$\text{mREE }\left(\text{kcal}/\text{day}\right)=\text{3}.\text{941}\times {\text{VO}}_{\text{2}}\left(\text{L}/\text{min}\right)+\text{1}.\text{1}0\text{6}\times {\text{VCO}}_{\text{2}}\left(\text{L}/\text{min}\right)-\text{2}.\text{17}\times \text{UN}\left(\text{g}/\text{day}\right),$$

where UN is the amount of nitrogen excreted in urine during 24 hours. The error in mREE if urine nitrogen is not measured is less than 2% [18].

**Figure 1:**
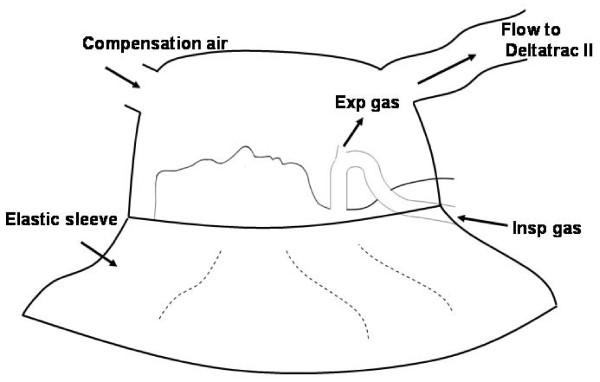
**Drawing of the measurement setup**. The head of the subject is covered with a transparent plastic half ellipsoidal canopy. A tight flexible plastic sleeve of the canopy is wrapped carefully under the pillow and around the inspiration tubing to minimize any leakages in the measurement circuit. The patient is ventilated with ambient air via the tracheostomy. Expiratory gases exit inside the canopy from the valve next to the tracheostomy.

Deltatrac II has two measuring modes: canopy and respirator modes. The canopy mode is intended, for measurements with spontaneously breathing patients. In that mode the inspiratory and excitatory gases are mixed under a half ellipsoidal plastic canopy. The respirator mode in turn is intended for measurements in mechanically ventilated patients. In that mode, the expired gases are usually collected through a mixing chamber to which the ventilator's expiratory tubing is connected [19]. We employed an open ventilation technique for our patients ie. the inspiration gases were conducted from the ventilator via a single inspiratory tube to the tracheostomy, but instead of a long expiratory tube, the expiratory gases were delivered to the room air via a valve near to the tracheostomy cannula. Due to this open ventilation technique we were not able to use the respirator mode in Deltatrac II and thus we used the canopy mode to collect all expiration gases during the measurements. The canopy was placed around the patient's head and the expiratory valve was placed inside the canopy. Any possible gas leakage from the measurement circuit was minimized using an elastic sleeve fixed to the canopy which was airtightly wrapped around the patient's upper body (as shown in Figure 1). To ensure appropriate measurements we compared two inflow settings for gas collection (Group Q_1 _= 40 L/min and Group Q_2 _= 80 L/min).

Before each measurement, the patient rested for a minimum of 30 min. Room temperature was between 20 and 24°C. Patients were asked to avoid any (even passive) physical activity such as physiotherapy for 10 hours before the measurement. During the measurement the patients were laying quietly in a supine position. The VO_2 _(mL/min) and VCO_2 _(mL/min) were measured once each minute. The mean total measurement time was 30 minutes for each measurement session. A stable period of a minimum of five minutes in mREE was considered as a valid measurement. The stability was determined as < 10% and < 5% deviations from the mean VO_2 _and RQ values, respectively. RQ values were required to be between 0.7 and 1.0. The patients' peripheral blood oxygen saturation was recorded constantly during the metabolic measurements with a portable non-invasive pulse oxymeter (Model 9845, Nonin, Minneapolis, USA).

Before each measurement and after a 30-min warming up period, the Deltatrac II was gas calibrated using an automatic procedure with a gas mixture containing 95% O_2 _and 5% CO_2_. Flow calibration was carried out every six months with the ethanol-burning test procedure. The accuracy of inflow value was ± 1.15% over the study period.

### pREE

The patients' pREE values were calculated using the Harris-Benedict [24], the Food and Agriculture/World Health Organisation/United Nation Union FAO/WHO/UNU [25], the Owen [26,27], the Mifflin-St Jeor [28] and the Fleisch equations [29] (Table 1). Because all patients had a long history of not being able to stand and consequent joint contractures, we used their heights at the time of diagnosis for the pREE calculations. The patients' weights were measured with a bed scale (ADE M60011, Germany). The body mass index (BMI) was calculated as BMI = weight (in kg)/height² (in cm).

### Laboratory analysis

Blood samples for determination of hemoglobin, glucose, plasma albumin, plasma prealbumin, plasma C-reactive protein and plasma thyroid hormone were taken. The body temperature was measured with a digital thermometer (Geratherm Medical, Germany). Increase in plasma C-reactive protein >10 g/L and body temperature > 37°C were considered for signs of infection. Aberrance from reference values in plasma thyroid hormone was considered as thyroid dysfunction. Low plasma albumin and prealbumin were considered as signs for malnutrition.

### Macronutritional intake

All patients were enterally fed daily via a percutaneous gastrostomy, but they were fasted 12 hours before the measurements. Only commercial nutrition substitutes (Nutrison Standard and Nutrison Multi Fibre, Nutricia Medical, Holland) were used. One patient was able to swallow small amounts of smashed food when assisted.

### Statistical methods

The results are given as mean ± SD if not mentioned otherwise. The effect of different flow values (Q_1 _and Q_2_) on mREE was analysed with Wilcoxon signed ranks test. The same test with Bonferroni's correction was used to compare mREE and pREE values calculated with five different equations. The level for statistical significance was considered as P < 0.05. The confidence intervals for the differences in medians were calculated (CIA statistical program version 2.1.2; Trevor Bryant, University of Southampton, UK). All other data were analyzed using the statistical program SYSTAT for Windows (version 10.2; Systat Software, Richmond, CA, USA).

## Results

The demographic characteristics of the patients are listed in Table 2. One REE measurement was rejected due to an infection. The mean given energy support was 1340 ± 150 kcal/d and the protein support was 0.8 ± 0.1 g/kg/d at the time of the measurements. The mean plasma albumin was 31 ± 4.0 g/L and prealbumin was 0.21 ± 0.1 g/L indicating normal nutritional status. All other blood chemistry results were within normal range as well.

**Table 2:** Characteristics of the five patients.

Variable	
Age (years)	55 (50 - 76)
Height (cm)	177 (155 - 192)
Weight (kg)	83 (58 - 98)
Body mass index (kg/m²)	25 (23 - 27)
Duration of the disease (months)	78 (64 - 122)
Duration in ventilator therapy (months)	78 (44 - 96)
Albumin (g/L)	31 (27 - 35)

Values are given as median (range).

The measured FiO_2 _was 20.9 ± 0.0% (ambient room air) in all measurements. The blood oxygen saturation for all patients was 97 ± 1% during all measurements. The values for mREE in Group Q_1 _(1110 ± 160 kcal/d) and in Group Q_2 _(1150 ± 200 kcal/d) were similar. We therefore pooled the mREE measurements from the Groups Q_1 _and Q_2 _for the comparison between mREE and pREE values. The mean mREE of all measurements was 1130 ± 170 kcal/d. When the mREE was compared with the mean pREE values calculated with all five different predictive equations, the mREE was 33.6% lower than pREE (*P *< 0.001) mREE (Table 3). All the predictive equations gave too high values for these patients. VO_2 _was 164 ± 23 and 165 ± 29 mL/min, VCO_2 _was 130 ± 23 and 142 ± 25 mL/min and RQ was 0.79 ± 0.1 and 0.87 ± 0.0 for Groups Q_1 _and Q_2_, respectively. The mean VO_2 _was 165 ± 25 mL/min, VCO_2 _was137 ± 24 mL/min and RQ was 0.82 ± 0.08 for all the measurements.

**Table 3:** Comparison of the values for measured (mREE) and predictive (pREE) resting energy expenditure in five patients having severe amyotrophic lateral sclerosis.

Equation	mREE (kcal/d)	pREE (kcal/d)	Difference between medians (95% confidence interval of the difference)
Harris Benedict [24]	1060 (960-1480)	1580 (1190-2020)	585* (425-760)

WHO/FAO/UNU [25]	1060 (960-1480)	1656 (1374-2039)	590* (472-780)

Mifflin-St Jeor [28]	1060 (960-1480)	1557 (1399-1909)	538* (439-688)

Owen [26,27]	1060 (960-1480)	1726 (1183-1879)	566* (414-691)

Fleisch [29]	1060 (960-1480)	1630 (1210-1938)	567* (417-727)

REE was measured by the use of indirect calorimetry on 13 occasions.

Values are median (range) or difference between medians (95% confidence interval of the difference). *P < 0.01 (Wilcoxon signed ranks test)

## Discussion

Our main finding is that the measured REE in mechanically ventilated ALS patients was 33.6% lower than that calculated with equations used generally for the prediction of REE. Even though our sample size of five patients is small, the results are very consistent and the difference compared to pREE values clear and they indicate that REE of ALS patients should be measured individually. The nutritional status of our patients who received nutritional support based on individual measurements was within the normal range, as indicated by their blood chemistry. However, the patient number is too small for making general conclusions concerning all those ALS patients who are permanently dependent on invasive ventilatory support.

Schimizu et al. measured, using indirect calorimetry, the REE of 11 ALS patients including also the thermal effect of food. These patients were chronically dependent on TIPPV with a mean duration of assisted ventilation of 4.6 years. Their energy expenditure was found to be 11.3-26.8% lower than that calculated with the Harris-Benedict equation which is well in accordance with our result. Schimizu et al. argued understandably that the decreased REE was due to ventilatory support and lack of the work of breathing [32]. Later, other authors have argued that instead of hypometabolism as evidently shown also in our study there would be hypermetabolism in severe stages of ALS. Kasarskis et al. measured both the REE and the forced vital capacity (FVC) in 16 ALS patients in the terminal stage of the illness. They found a positive correlation between decreased FVC and increased mREE and concluded that in terminal ALS patients the increased metabolic activity would be a consequence of greater breathing efforts needed to maintain life [33]. Desport et al. have also shown increased metabolic activity among ALS patients, but in contrast to Kasarskis' study they did not find a correlation between the decreasing values of lung function tests and increasing mREE values. However, they reported a correlation between an increased leucocyte count and increased mREEs and hypothesized that increased leucocyte activity may affect the cytokine secretion which, in turn, may enhance the production of free radicals leading to neuronal death [34,35]. These free radicals would also affect the mitochondrial activity leading to inappropriate heat production and increased metabolic activity [36]. However, in contrast to our ventilated patients and those of Schimizu et al. [32], a common feature of the studies by Desport et al. [34,35] and Kasarskis et al. [33] is that the ventilation of the patients was not artificially supported. Again our patients had normal BMI, nutritional status and albumin level indicating appropriate nutrition.

In healthy subjects, skeletal muscle tissue has been estimated to consume approximately 20-30% of total daily REE [37]. In patients with spinal cord injury the predictive equations have been found to overestimate the REE by approximately 5-32% depending on the level of the trauma [38,39]. The prevalence of an inappropriate weight gain and obesity is a common clinical problem for these patients, and it has been found in up to 60% among spinal cord injury patients [38-40]. Reduced muscle mass and metabolism together with low physical activity have been identified to cause the weight gain [38]. Our patient population included two subjects in a totally locked-in state and three with minimal movements in eyes indicating that they had lost all skeletal muscle tissue due to the progressive ALS. In addition, their ventilatory support was arranged with TIPPV. Our results support the hypothesis that reduced mREE is due to the atrophy of muscle tissue.

Deltatrac II is a device for indirect calorimetry and it can be used for REE determination both for spontaneously breathing as well as for mechanically ventilated subjects. It has been shown to have good overall accuracy. During mechanical ventilation the mean error is 2 ± 2% for VCO_2 _and 4 ± 4% for VO_2 _measurement. During spontaneous ventilation the mean error is 3 ± 2% for VCO_2 _and 4 ± 2% for VO_2 _measurement [20]. The canopy mode instead of the respirator mode of the device was used, because the TIPPV for our patients does include only a single tube for inspiratory ventilation gases, but not expiratory tubing from the tracheostomy to the ventilator. This kind of measurement setup may in theory expose the system to unwanted gas leaks from the canopy's inlet aperture. This, in turn, might cause too small measured VCO_2 _and VO_2 _values and therefore too small REE values, as calculated with the Weir's equation [31]. This may be possible if too small a sample gas flow is used. On the other hand, if too high a sample flow is used, there might be a risk for gas dilution which also could cause errors in measured VCO_2 _and VO_2 _values [18]. In order to eliminate these plausible errors two different sample flow settings (40 and 80 L/min) were used. Although the two flow settings produced similar VCO_2_, VO_2 _and mREE results, and although we took every measure to prevent gas leakages, we may have had a chance of a small systematic error.

The error for VO_2 _will increase if additional oxygen is used. This is due to the denominator of the Haldane equation (1- FiO_2_) which will decrease if too high a FiO_2 _is used. Also, the smaller the FiO_2 _is, the smaller an error in measured VO_2 _is plausible [18]. Errors due to additional oxygen were minimized in the present study, as we did not give any supplemental oxygen during the measurements.

## Conclusion

Our results show a significantly decreased REE in ALS patients who are chronically dependent on TIPPV. Because ALS patients may live years with the support of TIPPV [8,11], their nutritional support should also be individualized and based on true measurements. Nevertheless, our results should be interpreted with caution, until verified in a larger patient population.

## Competing interests

The authors declare that they have no competing interests.

## Authors' contributions

All authors have participated in conception and design of the study, analysis and interpretation of the data, drafting of the article and critical revision of the article for important intellectual content, and approved the final version of the article.
